# Image-Based Assessment of Drought Response in Grapevines

**DOI:** 10.3389/fpls.2020.00595

**Published:** 2020-05-15

**Authors:** Nunzio Briglia, Kevin Williams, Dan Wu, Yaochen Li, Sha Tao, Fiona Corke, Giuseppe Montanaro, Angelo Petrozza, Davide Amato, Francesco Cellini, John H. Doonan, Wanneng Yang, Vitale Nuzzo

**Affiliations:** ^1^Dipartimento delle Culture Europee e del Mediterraneo, Università degli Studi della Basilicata, Matera, Italy; ^2^National Plant Phenomics Centre, IBERS, Aberystwyth University, Aberystwyth, United Kingdom; ^3^National Key Laboratory of Crop Genetic Improvement and National Center of Plant Gene Research, Huazhong Agricultural University, Wuhan, China; ^4^ALSIA, Centro Ricerche Metapontum Agrobios, Metaponto, Italy

**Keywords:** greener fraction, leaf angle, Multi-view stereo, plant phenotyping, *Vitis vinifera*, water stress, 3D imaging

## Abstract

Many plants can modify their leaf profile rapidly in response to environmental stress. Image-based data are increasingly used to retrieve reliable information on plant water status in a non-contact manner that has the potential to be scaled to high-throughput and repeated through time. This paper examined the variation of leaf angle as measured by both 3D images and goniometer in progressively drought stressed grapevine. Grapevines, grown in pots, were subjected to a 21-day period of drought stress receiving 100% (CTRL), 60% (*IRR*_60%_) and 30% (*IRR*_30%_) of maximum soil available water capacity. Leaf angle was (i) measured manually (goniometer) and (ii) computed by a 3D reconstruction method (multi-view stereo and structure from motion). Stomatal conductance, leaf water potential, fluorescence (*F*_*v*_/*F*_*m*_), leaf area and 2D RGB data were simultaneously collected during drought imposition. Throughout the experiment, values of leaf water potential ranged from −0.4 (CTRL) to −1.1 MPa (*IRR*_30%_) and it linearly influenced the leaf angle when measured manually (*R*^2^ = 0.86) and with 3D image (*R*^2^ = 0.73). Drought was negatively related to stomatal conductance and leaf area growth particularly in *IRR*_30%_ while photosynthetic parameters (i.e., *F*_*v*_/*F*_*m*_) were not impaired by water restriction. A model for leaf area estimation based on the number of pixels of 2D RGB images developed at a different phenotyping robotized platform in a closely related experiment was successfully employed (*R*^2^ = 0.78). At the end of the experiment, top view 2D RGB images showed a ∼50% reduction of greener fraction (GGF) in CTRL and *IRR*_60%_ vines compared to initial values, while GGF in *IRR*_30%_ increased by approximately 20%.

## Introduction

Plants constantly adapt to their changing surroundings, adjusting their physiology, development and growth (Schurr et al., 2006). This dynamic adaptation can have both long-term (weeks to months) effects (e.g., shoot elongation, total leaf area development) as well as short-term effects (minutes to hours) that include changes in foliage orientation and leaf temperature (Biskup et al., 2007). Monitoring adaptations triggered by the changing environment are relevant for appropriate choice of management actions and in breeding programs. For example, monitoring the variations of trunk diameter or leaf turgor in response to soil moisture oscillations can direct an irrigation schedule (Goldhamer and Fereres, 2001; Jones, 2006). To monitor these traits, ideally one should use non-contact and non-destructive sensors (e.g., thermocouples and/or thermistors, leaf turgor pressure, trunk diameter gauges, dendrometer, strain gauges) (Ortuño et al., 2010; Fernández, 2014) as these have the potential to be automated and scaled at lower cost than manually implemented approaches.

Image-based sensors have become cheaper, more robust and are intrinsically non-contact. Information derived from images has the potential to parameterize ecophysiological models and predict the impact of environmental factors on various plant and fruit traits including growth, diseases incidence and chemical composition (Gago et al., 2017; Zhao et al., 2019). Drought events are predicted to be more frequent and longer in coming decades in many cultivated regions (e.g., Mediterranean) (Raymond et al., 2019), so image-based assessment of drought stress in crop species are urgent (Berger et al., 2010; Briglia et al., 2019).

Leaf angle is a key indicator of water relations in grapevine mainly because angle changes according to water status or turgor (Smart, 1974). Change of leaf angle has also been implicated in water stress tolerance. For example, leaf angle variation can reduce the thermo-radiative load on leaf (and in turn its temperature, conductance and transpiration), minimizing the inhibition of photosystem II and contributing to water conservation (Gamon and Pearcy, 1989; Palliotti et al., 2008). Many grass species also display leaf blade rolling in response to drought, reducing the surface exposed to thermal radiation and such responses are quantifiable using image based analyses (Duan et al., 2018).

Non-contact sensing of the plant and the environment is increasingly important for crop irrigation management strategies, saving water while maintaining fruit quality (Fernández, 2017; Khanna and Kaur, 2019). Leaf angle is receiving increasing attention within 3D modeling of water dynamics at both leaf and ecosystem scales, due to its influence on water transport and reflectance/absorbance processes in several species (Vicari et al., 2019) including grapevines (Zhu et al., 2018). Multi-view stereo (MVS) 3D reconstruction is increasingly employed to generate 3D point clouds for reconstruction of plants, canopies and estimation of diverse traits such as main shoot height, fruit and leaves in tomato, maize, rape (Lou et al., 2014; Xiong et al., 2017). Various 3D models of plant canopy reconstruction for reliable drought stress characterization also based on MVS photogrammetry have been recently compared (Srivastava et al., 2017; Das Choudhury et al., 2019) but the approach has not yet been applied to grapevines.

Although leaf angle or leaf inclination relative to shoot is related to leaf water potential in several crops (e.g., soybean, wheat, pepper, prune, apricot) (Kao and Forseth, 1991; Torrecillas et al., 2000; Lampinen et al., 2004), it is not yet a common parameter in irrigation management, likely because of practical and cost constraints in handling leaves and measurement. Hence, improved estimation of leaf angle would be highly desirable to potentially improve smart management of irrigation.

A combination of 2D and 3D imaging of specific plant structure/architecture features that respond to drought stress (e.g., leaves orientation, grains and fruits number and structures, primary and secondary roots distribution) could provide smart tools for digital agriculture. For example, in soybean subjected to water deficit, a combination of 3D laser scanning and stereovision reconstruction of leaf revealed the spatial orientation of single leaf and in turn quantified drought stress (Biskup et al., 2007). Point clouds derived from 3D laser scanning have been proposed for leaf and stem classification in grapevine (Paulus et al., 2013) but leaf angle was not tested.

In grapevine, the relationship between manual and image-based measurements of leaf angle and leaf water potential is unknown. It has been reported that manually measured leaf angles might change from the vertical axis by 60–70° in well irrigated vines up to 80–120° in drought stressed ones when approaching severe drought condition (approximately −1.8 MPa early morning) (Smart, 1974; Palliotti et al., 2008; Briglia et al., 2019). However, a systematic characterization of leaf angle response to drought stress using 3D image-based plant-phenotyping domain has not been adequately explored.

Therefore, this study mainly aimed at comparing the variations of leaf angle as measured using 3D images with those using manual (goniometer) methods in progressively drought stressed grapevines.

## Materials and Methods

### Plant Material and Experimental Design

The experiment was conducted at the National Plant Phenomics Centre, IBERS-Aberystwyth University, United Kingdom (N 52° 24′ E -4° 01′) during the 2018 growing season in a greenhouse with controlled environmental conditions. The minimum air temperature was set at 18°C and active radiation (PAR) at approximately 800 μmol m^–2^ s^−1^ (natural light supplemented with 600 W sodium lamps) from 0500 to 2000 h. Air temperature and relative humidity were recorded at 5 min interval through the greenhouse integrated wireless sensors (Cambridge sensors)^1^, the vapour-pressure deficit (*VPD*) was then automatically computed following Goudriaan and van Laar (1994).

A total of 45 vines (cv Aleatico) grafted on 110R rootstock were grown in black 3.5 L PVC pots filled with a 3:1 v/v mixture of sandy loam soil (82% sand, 7% silt, and 11% clay) and Levingtons F2 peat compost. Maximum soil available water capacity (AWC) (g) was calculated following Minasny and McBratney (2003). The reference soil weight (g) at the field capacity (FC) was determined on 5 soil samples collected after fully irrigating the pots and allowing water to drain for 15 h, until a stable weight was reached. Then the soil weight at the permanent wilting point (WP) was obtained by drying the soil samples at 80°C until a stable weight was reached. The AWC was calculated as difference between FC and WP (Minasny and McBratney, 2003).

The potted vines were placed on a gravimetric platform, composed of a set of precision scales (± 1g) equipped with a computer controlled irrigation system^2^. Based on the pot weight recorded at 1800 h the platform computed the daily water consumption (g) per vine in each irrigation group as the difference between the reference soil weight and actual plant weight.

From potting of the cuttings (15^th^ of March) until the beginning of irrigation treatment (see below) plants received every 15 days 150 ml plant^−1^ of an aqueous solution containing 3 g L^−1^ of Chempack Low Nitrogen Feed Fertilizer (NPK 12.5-25-25).

### Irrigation and Drought Stress Imposition

From bud-break (09 BBCH-scale) (early April) till the imposition of irrigation treatments (55 BBCH-scale) (i.e., 21^st^ of May, hereafter referred as “Day 0”), all vines were fully irrigated just after they were weighed replacing 100% of the amount of daily water consumption to keep soil moisture close to field capacity. At Day 0, 15 vines were allocated to each of 3 irrigation treatments (fraction of water to be replenished via irrigation): restoring 100% (control, CTRL), 60% (*IRR*_60%_) and 30% (*IRR*_30%_) of the AWC.

### Stomatal Conductance and Chlorophyll-*a* Fluorescence

**FIGURE 1:**
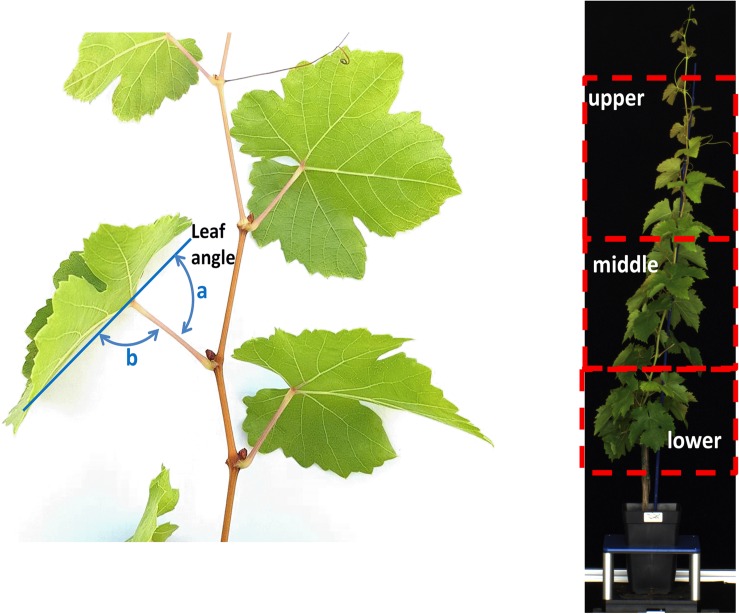
Left: schematic diagram of the protocol used to determine (a) leaf angle as supplementary of (b) angle of midrib from petiole; right: representation of the lower (nodes 1–5 from ground), middle (nodes 6–11) and upper (nodes >11) region of a plant canopy.

Stomatal conductance (*g*_*s*_) per unit leaf area was measured midday (1130–1230 h) at −3, 0, 2, 3, 8, 10, 14, 16, and 21 days after treatment imposition (DADI) using a portable porometer (Delta-Device ΔP4). The measurements were performed on 4–5 vines per irrigation treatment on two fully expanded leaves per vine selected from the middle region of plant canopy (nodes 6–11 from the ground, see Figure 1).

On the same leaf used for *g*_*s*_ measurements, chlorophyll *a* (Chl-*a*) fluorescence (*F*_*v*_/*F*_*m*_) (see Briglia et al., 2019 and reference therein) was measured at midday (1130–1230 h) through a portable chlorophyll fluorometer (PAM- 2500, Heinz Walz GmbH, Effeltrich, Germany).

### Stem Water Potential

At 0, 3, 10, 14, 16, and 21 DADI the stem water potential (Ψ) was measured midday using a pressure bomb (The SKPM 1400 series, Skye Instruments Llandrindod Wells Powys, United Kingdom) following the methodology reported by Turner (1981). On each measurement time, 2 leaves per pot (×3–4 pots a treatment) from the middle canopy region (Figure 1) were used.

### Soil Moisture

Soil moisture was determined gravimetrically at the end of each *g*_*s*_, Chl-*a* and Ψ sampling session. Soil samples were taken from each pot per treatment (*n* = 3–4), weighed (FW, fresh weigh) and dried till constant weight (DW). Soil moisture was calculated as (FW-DW)/DW × 100 and reported as %DW, according to Black (1965).

### Leaf Angle and HTP Plant-Phenotyping

After the physiological measurements were collected, the same individuals were used for various image acquisitions.

#### Leaf Angle

Leaf angle was defined as the supplementary angle of the deviation angle of the midrib from petiole according to Smart (1974; Figure 1). At 0, 3, 10, 14, 16, and 21 DADI, the leaf angle was manually measured using a goniometer on 8 leaves per vine (×3–4 plants a treatment) selected from the lower (nodes 1–5 from ground), middle (nodes 6–11) and upper (nodes >11) region of the canopy (Figure 1).

**FIGURE 2:**
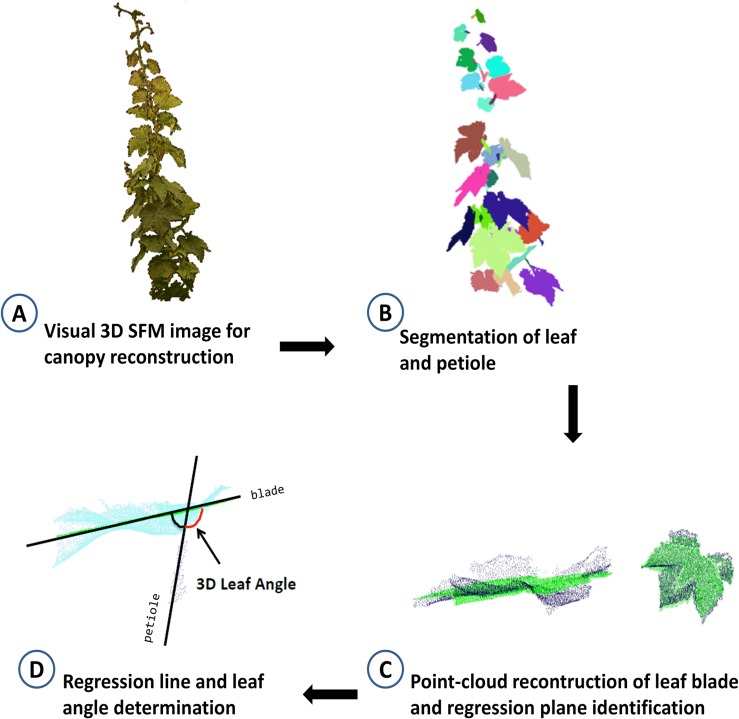
Schematic work-flow of the procedure for 3D identification of leaf angle showing **(A)** a digital plant canopy obtained using 60 side-view images and structure from motion reconstruction (SFM) algorithm to obtain 3D cloud points; **(B)** leaf and petiole segmentation after removing noise points, region growing, filling small holes, the leaf and petiole were segmented semi-automatically; **(C)** determination of leaf blade regression plane and petiole regression line; **(D)** calculation of the leaf angle.

Leaf angle was also determined through a multi-view stereo (MVS) 3D reconstruction method of plants using multi-view images to feed a Structure-From-Motion procedure followed by a stereo matching and depth-map merging process (Wu, 2013; Lou et al., 2014; Xiong et al., 2017). At 0, 2, 3, 8, 9, 14, 16, and 21 DADI, vines were imaged at the same time of day (between 1200 and 1300 h) using a consumer grade color camera (Nikon Reflex Camera, 6,000 × 4,000 pixel resolution). The 3D imaging station (GreenPheno, Wuhan, China) also included a tripod, a rotatable platform and a servomotor controller, controlled by the image acquisition software (written in LabVIEW). Diffuse illumination was provided by 2 150 W halogen lamps (Patterson TL 3200K, United Kingdom). The plants were imaged by placing on the rotatable platform that was programed to stop every 6°, allowing the capture of 60 images per vine. All images (.PNG) were stored in a database (raw data available on request) and subsequently obtain the 3D point cloud with visual structure from motion (SFM) algorithm. Then, using the 3D model of each vine, the leaf angle was calculated using Cloudcompare and point cloud library (PCL). Figure 2 summarizes the main steps for leaf angle estimation from 3D models: (A) SFM reconstruction algorithm using 60 side-view images to obtain 3D cloud points; (B) identification and removal of noise points (see below), region growing, fill small holes, semi-automatic segmentation of leaf and petiole; (C) point-cloud reconstruction of leaf blade and identification of regression plane; (D) determination of petiole regression line and calculation of the 3D leaf angle.

The identification of noise points was based on the gray-values (r, g, and b) of RGB color channels of each point, then the removal was executed when that values were below thresholds according with the following criteria:

r+g+b≤70⁢or⁢g/b≤1.

#### RGB Imaging and GGF Index

Vines were also imaged (between 1200 and 1300 h) at 0, 2, 3, 8, 9, 14, 16, and 21 DADI using a LemnaTec Scanalyzer phenotyping platform (LemnaTec GmbH, Aachen, Germany). Vines were automatically conveyed into an imaging chamber equipped with a visible light (RGB) sensor with a 2454 × 2056 pixel resolution. Four images were acquired per vine: a Top View (TV) image taken from above and 3 Side Views (SV) taken at 0°, 45° and 90°. Image analyses were performed using the software LemnaGrid v2 following Zaman-Allah et al. (2015) and the segmentation procedure reported in Briglia et al., 2019.

RGB images were then converted to HIS (hue, intensity, saturation) color space. Next, the component H (hue, in degree) was used to calculate the “greener fraction” (GGF) as the number of green pixels (80° < hue < 180°) relative to the total number of pixels of a given image (Casadesús and Villegas, 2014).

### Leaf Area Determination

The projected shoot area (PSA) was calculated from the 2D RGB images, according to Briglia et al. (2019) and modified as follows:

(1) $$\text{PSA}=N_{\text{pix}}\,0^{\circ }\,\text{SV}+N_{\text{pix}}\,45^{\circ }\,\text{SV}+N_{\text{pix}}\,90^{\circ }\,\text{SV}\;+0.3\times N_{\text{pix}}\,\text{TV}\,\left(p\,i\,x\,e\,l\right)$$

where “*N*_pix_ 0°SV,” “*N*_pix_ 45°SV” and “*N*_pix_ 90°SV” and “*N*_pix_ TV” is the number of pixels corresponding to the plant object area of the images collected at the various positions.

After final image acquisition, the vines were manually defoliated, leaves were scanned on a flat bed scanner and the leaf area (*LA*, cm^2^) measured using Fiji open source software (Schindelin et al., 2012; Maloof et al., 2013).

### Data Analysis

The statistical analysis used R software (3.3.2 version) package agricolae” (de Mendiburu, 2016), plotting and fitting were by OriginPro 9.3 (OriginLab Corporation, United States). Data were reported as mean and standard error of the mean (±SE). A one-way ANOVA was used to examine the differences between irrigation treatments at each sampling date, the differences among means were identified by Tukey Honest Significance Difference (HSD) *post hoc* tests and *p* values < 0.05 were considered significant.

## Results and Discussion

### Plant Water Relations and Environmental Conditions

This study focused mainly the response of leaf angle (as measured using either a goniometer and through a 3D image-based process) to drought stress in grapevines experiencing a relatively wide range of Ψ (see below). Vines were grafted on 110R rootstock, which is a common rootstock used in dry environments to deal with limitation of soil water content due to its structure and function (Yıldırım et al., 2018).

The change of leaf angle in grapevine canopy is a turgor response of the plant to the reducing soil available water (Smart, 1974). However, it has been also documented in *Vitis* spp. that variation of leaf angle mediates the trade-off between the need for carbon gain and for avoidance of excessive radiation load (Gamon and Pearcy, 1989). In this study grapevines grew at saturating light condition (800 μmol m^–2^ s^−1^ PAR) (Greer and Weedon, 2011) to prevent any effect of excessive irradiance load on photoinhibition and in turn on leaf angle (Gamon and Pearcy, 1989). Hence, leaf angle variations here presented are attributable only to plant water status *sensu* Smart (1974).

**FIGURE 3:**
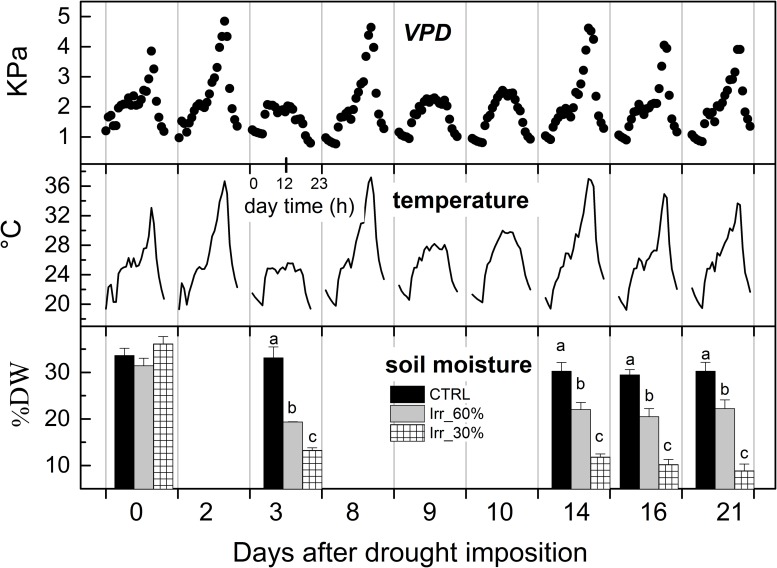
Diurnal variations of vapor pressure deficit (*VPD*) and air temperature recorded inside the glasshouse during the experiment and average values (± SE) of soil moisture (% dry weight) measured across the experiment in vines receiving 60% (*IRR*_60%_) and 30% (*IRR*_30%_) of the available water capacity and under well irrigation (CTRL). For soil moisture, comparing treatments at the same time different letter indicates statistically significant differences (Tukey’s HSD, *p* < 0.05), letters were not reported when differences were not significant.

During the experiment, values of maximum *VPD* ranged from approximately 0.6 (12 DADI) to 4.9 kPa (2 and 8 DADI), with an average value of 2 kPa, and a mean air temperature value of 25.5°C (Figure 3). Soil moisture in well irrigated pots was stable at approximately 32% DW throughout the experiment while it gradually reduced in drought stressed ones starting from 3^rd^ DADI (Figure 3) and approaching values close to 10% DW (*IRR*_30%_), and 22% DW (*IRR*_60%_) at the end of the experiment (Figure 3). Changes of soil moisture are comparable to that reported in a closely related experiment carried out at a different robotized plant-phenotyping facility (Briglia et al., 2019).

**FIGURE 4:**
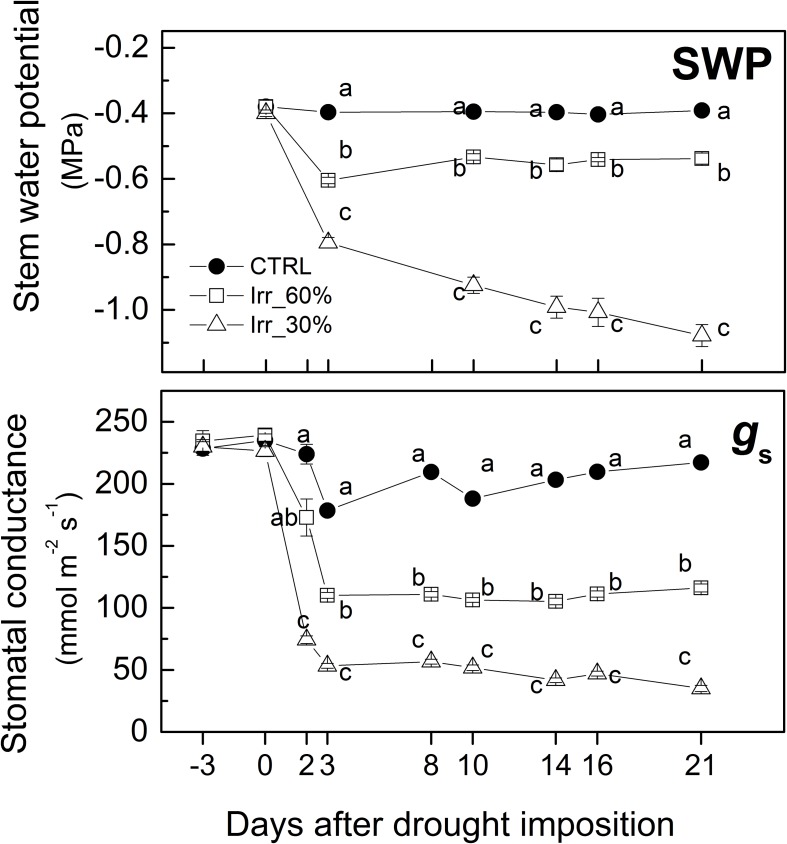
Pattern of mean values (±SE) of stem water potential (SWP) and stomatal conductance (*g*_*s*_) measured midday in leaves of grapevines under drought stress receiving 60% (□, *IRR*_60%_) and 30% (Δ, *IRR*_30%_) of the available water capacity and well watered (•, CTRL). Comparing treatments at the same time different letter indicates statistically significant differences according to Tukey’s HSD test, *p* < 0.05. Note that letters were not reported when differences were not statistically significant.

Although pre-dawn leaf water potential is among the most accurate parameter for plant water status assessment (Chone et al., 2001) in this study stem water potential was measured at midday because of its close correspondence with that measured pre-dawn (Chone et al., 2001). Throughout the experiment, the Ψ values of the CTRL vines were stable at approximately −0.4 MPa (Figure 4) similarly to Shackel (2007) and Giorio and Nuzzo (2012). In vines under moderate drought stress (*IRR*_60%_) Ψ declined close to −0.6 MPa at 3 DADI where it remained throughout the experiment (Figure 4). In *IRR*_30%_ vines the values of Ψ decreased to approximately −0.8 MPa after 3 days of drought stress, thereafter it progressively declined to about −1.1 MPa at 21 DADI (Figure 4).

Stomatal conductance was measured also before drought stress imposition (at −3 DADI) to check for vines uniformity showing values at approximately 240 mmol H_2_O m^–2^ s^−1^ (Figure 4) similarly to that of field grown grapevines (Chaves et al., 2010). Next days and until 3 DADI, the stem water potential decreased exerting the down-regulation of stomatal closure in both *IRR*_60%_ and *IRR*_30%_ (Figure 4) similarly to observations reported for pot and open-field studies (Medrano et al., 2003 and Cifre et al., 2005). In *IRR*_60%_ *g*_*s*_ reduced to a value approximately 50% of the initial one as soon as 3 DADI, it stayed there for the remaining part of the experiment (Figure 4). In *IRR*_30%_ vines values of *g*_*s*_ were ∼70% lower than that of CTRL vines at 3 DADI, thereafter it continued to decline toward the minimum at 14 DADI where it remained until the end of the experiment (Figure 4).

As anticipated, the present experiment was designed to avoid possible influence of (excessive) leaf irradiance load on its angle preventing occurrence of photoinhibition. The values of *F*_*v*_/*F*_*m*_ measured across −0.4 to −1.1 MPa range of Ψ remained optimal (i.e., close to 0.75) (not shown) similarly to Briglia et al. (2019) where drought was combined with high irradiance (PAR at approximately 2,000 μmol m^–2^ s^−1^). Hence, in addition to drought protective mechanism(s) recognized in grapevine (e.g., improved photosynthetic indexes, increased in carotenoids, ROS, proline) (Medrano et al., 2003, Carvalho et al., 2015), leaves of the Aleatico cv might have a cultivar-specific compensatory mechanism(s) of the photosystem (e.g., augmented electron transport capabilities) (Hochberg et al., 2013).

### Leaf Angle Response to Drought Stress

**FIGURE 5:**
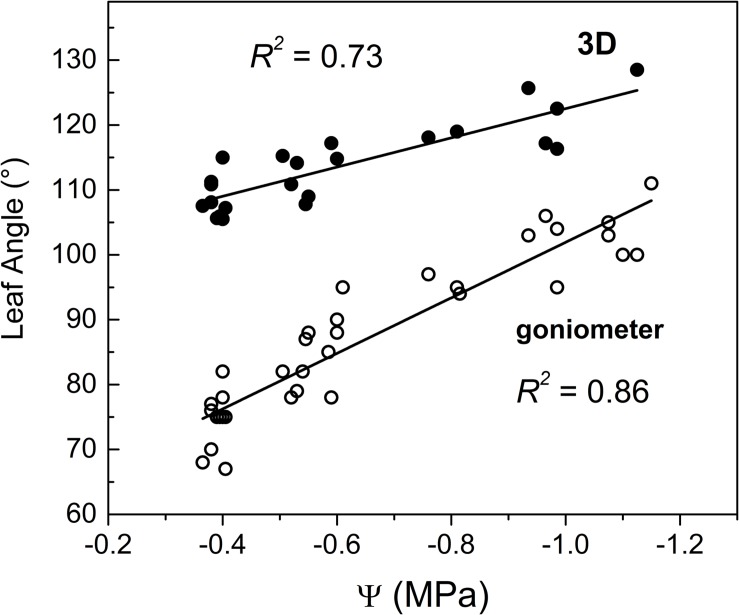
Correlation between stem water potential measured midday (Ψ) at the middle region of canopy and leaf angle measured through (□) goniometer and (•) 3D-image method. Note that goniometer measurements refer to the middle canopy zone while 3D leaf angles are the average of 3–15 measurements collected in various plant canopy zones.

Results reveal a linear relationship between Ψ and leaf angle as measured with both 3D and manual methods. Leaf angle (goniometer) gradually increased from approximately 75° (well irrigated) to approximately 110° (severely drought stressed) (Figure 5). The present results are difficult to compare with the existing literature because of poor existing data on leaf angle across a wide range of Ψ. However, results are in line with that of Palliotti et al. (2008) who reported a variation from 76° (well irrigated) to 88° (water stress) but, unfortunately values of related leaf water potential were not reported so preventing a deeper discussion. However, the approximately linear increase of leaf angle in response to decreasing Ψ (Figure 5) fits quite well with results from drought stressed soybean (Kao and Forseth, 1992).

**FIGURE 6:**
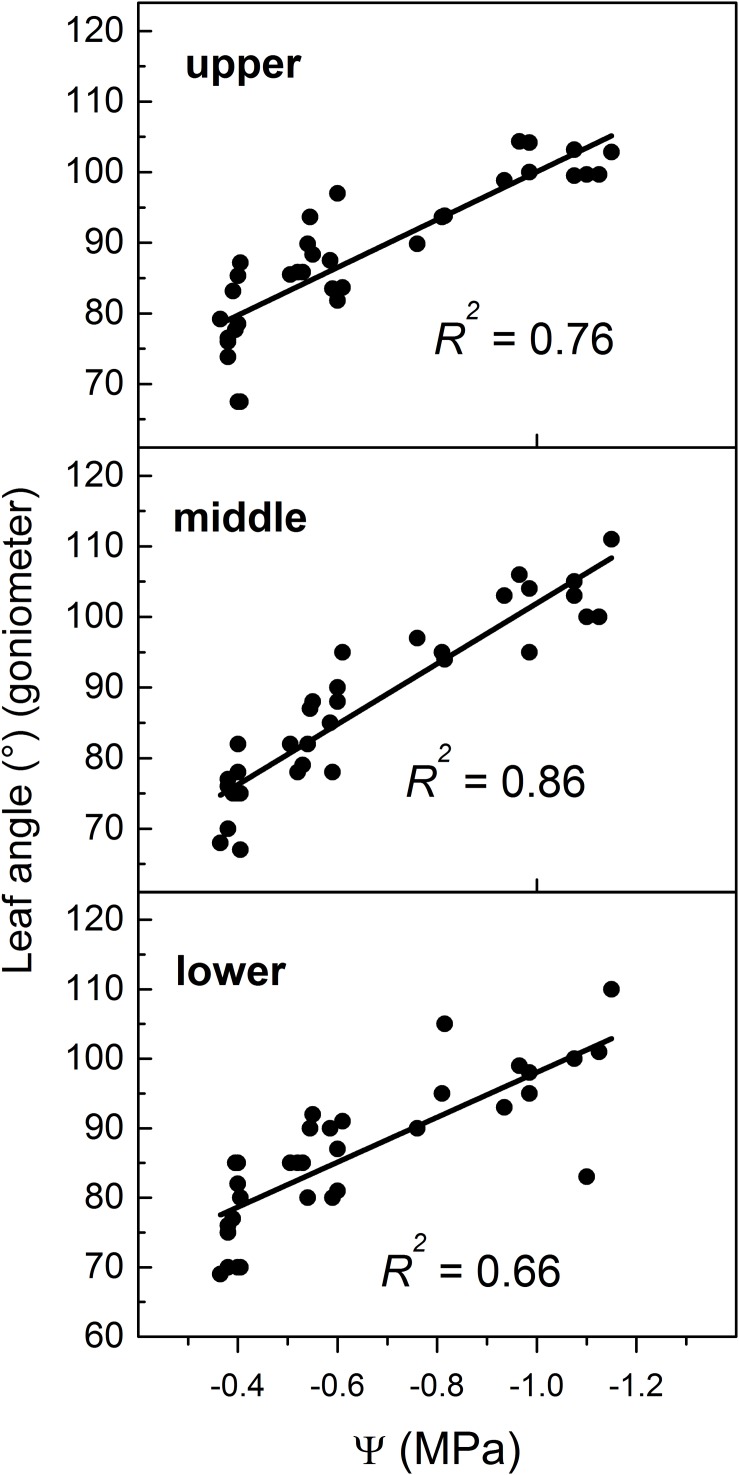
Relationship between leaf angle manually measured at three regions (upper, middle, and lower) of the canopy and midday leaf water potential (Ψ). Note that in all panels values of Ψ refer to the Ψ measured in the middle region. For location of regions please refer to Figure 1.

In this study, the Ψ was measured in the middle zone of the canopy while leaf angle was manually measured at the three zones identified (i.e., lower, median and upper) (Figure 1). Hence, leaf angle showed a variable dependence on Ψ according to the canopy region where the angle was measured (Figure 6), which might reflect the variable hydrostatic pressure gradient existing across the vine (Zhu et al., 2018). However, more efforts are required to examine heterogeneity of leaf angle through the canopy as influenced by Ψ gradient.

This study reports 3D image-based data on specific drought-related trait in grapevine (leaf angle) integrating current information (Srivastava et al., 2017; Das Choudhury et al., 2019).

Leaf angles were gentler when measured using the 3D method for a given Ψ compared to angles measured with the goniometer (Figure 5). Namely, the manual and 3D methods differed by approximately 15–20° in severely water stressed vines (i.e., Ψ close to −1.1 MPa) while differences increased up to 35° in well irrigated vines (i.e., Ψ close to −0.4 MPa). The higher accuracy of leaf angle measurement intrinsically conferred by its direct (manual) and careful determination (Norman and Campbell, 1989) might help to explain its higher coefficient of determination compared to that obtained through the 3D method.

The 3D image-based procedure employed determines the leaf angle as an average of those angles identified in various canopy regions while Ψ was measured in a specific region (see Figure 6). This might help also to explain differences detected between the two methods and the relatively lower predicting strength of the 3D method compared to that of goniometer.

**FIGURE 7:**
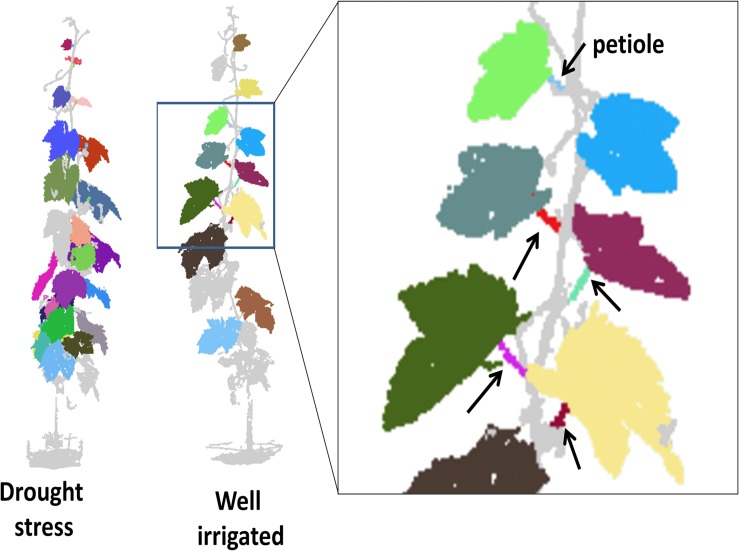
Schematic output of the 3D point cloud reconstruction and organ segmentation for a drought stressed (Ψ value approximately –1.0 MPa) and well irrigated (approximately –0.4 MPa) vines imaged at 14 DADI. On the right, the partial enlargement of the well irrigated canopy shows the identified petioles (arrows). Note that different colors indicate different items identified; colors apply only for electronic version of the article.

The 3D point cloud data analysis allowed the segmentation of leaf blade planes and petioles as shown by the animated visualization of the 3D vine (Supplementary Video S1) and by Figure 7. However, the number of petioles successfully segmented in each vine (and in turn that of leaf angles determined) was lower (3–15) as compared to the actual number of leaves present (20–25) because the 3D point-cloud reconstruction was sensitive to overlapping or occluded leaves. The topographical position of identified angles around the canopy was not recorded, contributing to reduced predictive accuracy of the 3D method as compared to goniometer.

Bailey and Mahaffee (2017) developed a method based on terrestrial LiDAR scanning data to estimate the distribution of leaf orientation for an arbitrary volume of *Vitis vinifera* and *Poplus balsamifera* leaves, overcoming some of the occlusion issues. In that study, the reduction of sampling volume and multiple scanning potentially fix the overlapping issue. For laboratory experiments, the vines might be trained to a simpler architecture to minimize leaf overlapping (e.g., single shoot or shoots well outdistanced). To address the erratic identification (number and position) of leaves using the 3D method for the estimation of the leaf angle in grapevine requires more effort.

The methods adopted in this study required the segmentation of both the lamina and the petiole for the same leaf to calculate the leaf angle. In a recent study the inclination of leaf surface relative to the zenith was determined through terrestrial LiDAR point clouds without accounting for the petiole (Vicari et al., 2019). Leaves of the tree species studied (e.g., *Ostrya japonica*, *Diospyros lotus*, *Ginkgo biloba*, *Wollemia nobilis*) are sessile or have a shorter petiole as compared to that of *Vitis* spp., however it would be worth testing whether that method is applicable to species with long petiole as grapevine. In addition, in *Vitis* spp. the inclination of leaf blade *per se* (and in turn leaf angle) might be influenced by several factors not directly related to plant water status (e.g., shoot position, row orientation, training systems) (Louarn et al., 2007). Hence, measuring changes of leaf angle relative to petiole is essential to avoid confounding factors if plant water status is to be determined. This study mainly related the computer vision signal to the underlying physiology (i.e., leaf angle), being potentially supportive for development and exploitation purposes of a new 3D-image based method of Ψ assessment within precision agriculture domain.

### RGB-Image Based Morphometric and Colorimetric Indexes

Several leaf traits (e.g., orientation, thickness, pigments content, trichomes, stomatal conductance, photosynthetic rate) contribute to drought tolerance in plants and are often reinterpreted within a HTP-phenotyping context (Berger et al., 2010). Trichome density of the abaxial surface is a cultivar specific trait varying from “absent” to “very dense” (IPGRI et al., 1997), and a dense trichome layer may confer a lighter green color compared to the a gluacous adaxial leaf surface (Boso et al., 2010). On that basis, it was previously hypothesized that a reduction of dark green fraction occurs in leaves of drought stressed grapevine because of the increased exposure of the lighter green abaxial leaf surface due to the increased leaf angle (Briglia et al., 2019). The Aleatico cv has no (or very sparse) trichomes (Organisation Internationale de la Vigne et du Vin [OIV], 2009, Paolocci et al., 2014) so that hypothesis is not plausible. In addition the color of Aleatico young leaves as discussed above poses further issues. However, it would be worth to test it in case of varieties with higher trichome density.

**FIGURE 8:**
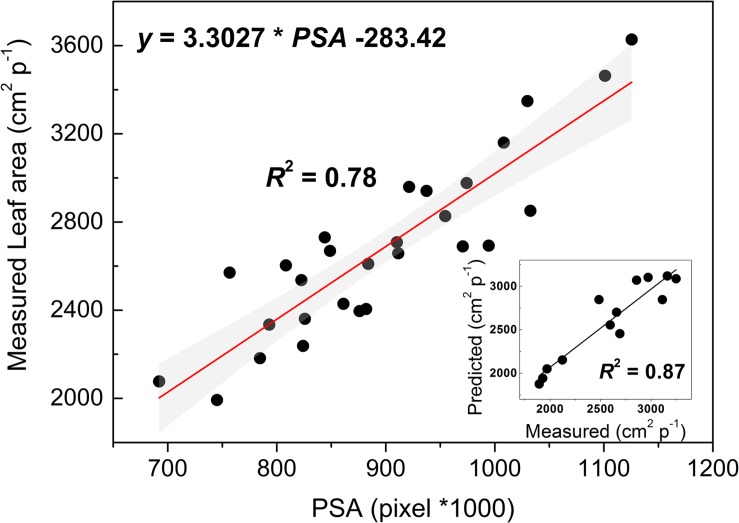
Linear fitting model of leaf area (*y*-axis) and the projected shoot area (PSA) resulting from a 10-fold cross-validation analysis. The gray filled area indicate the upper and lower 95% CI about the model. In the inset, a regression analysis testing the model on a different set of vines.

Vine development, as influenced by drought imposition, was assessed through the estimation of change in leaf area (*LA*’) employing an RGB-images based model. The linear *LA*’ model (*LA*’ = 3.3027 × *PSA* −283.42, *R*^2^ = 0.78) (Figure 8) was trained through a resampling (10-fold) Cross-Validation procedure, revealing its good performance (*R*^2^ = 0.87) in predicting *LA*’ of new test data. This was substantially confirmed by testing the model on a set of 13 additional vines (see the inset of Figure 8). Values of intercept and slope of the *LA*’ model differed from that of the model developed and validated for well irrigated and droughted grapevines at another robotized HTP plant-phenotyping platform located in Southern Italy (Briglia et al., 2019). Such differences might conceivably be explained considering the different RGB camera resolution of the two HTP facilities and the inclusion of the 45°SV pixels into the equation for PSA determination. Noticeably, addition of the 45°SV pixels allowed a model performance as high as in Briglia et al. (2019) likely because of higher canopy density. Lack of comparable standards and protocols across platforms is a critical issue for the phenotyping community (Reynolds et al., 2019; Rosenqvist et al., 2019). Hence, the present results might support the harmonization and standardization of protocols among HTP facilities (Reynolds et al., 2019).

**FIGURE 9:**
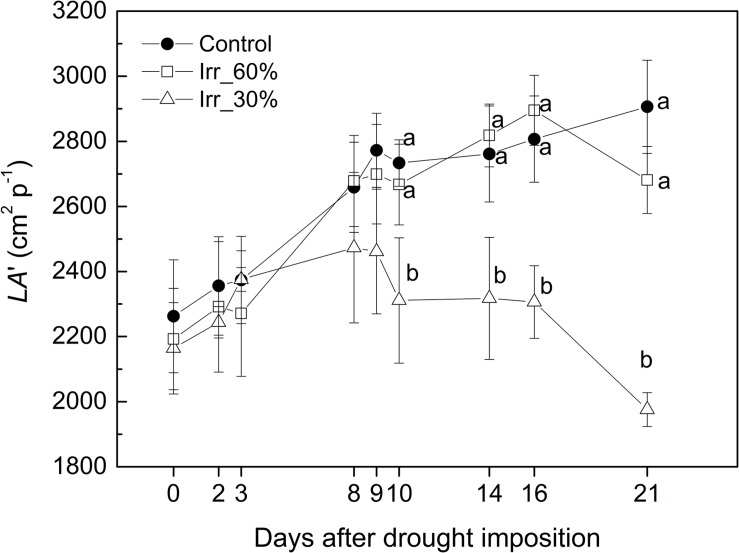
Evolution of the estimated leaf area (*LA*’) in grapevines under drought stress receiving 60% (□, *IRR*_60%_) and 30% (Δ, *IRR*_30%_) of the available water capacity and well watered (•, CTRL).

Leaf area of CTRL and *IRR*_60%_ vines increased by approximately 26% within the experiment reaching approximately 2,800 cm^2^ p^−1^ (Figure 9). For the *IRR*_30%_ vines leaf area showed an initial 14% increase during the early 9 DADI, then it remained stable for a week before a final decline toward the lowest value of 1,970 cm^2^ p^−1^ (21 DADI) likely due to an initial defoliation triggered by drought stress. The influence of the irrigation treatment on leaf area growth estimated through *LA*’ is consistent with Campo et al. (1999).

**FIGURE 10:**
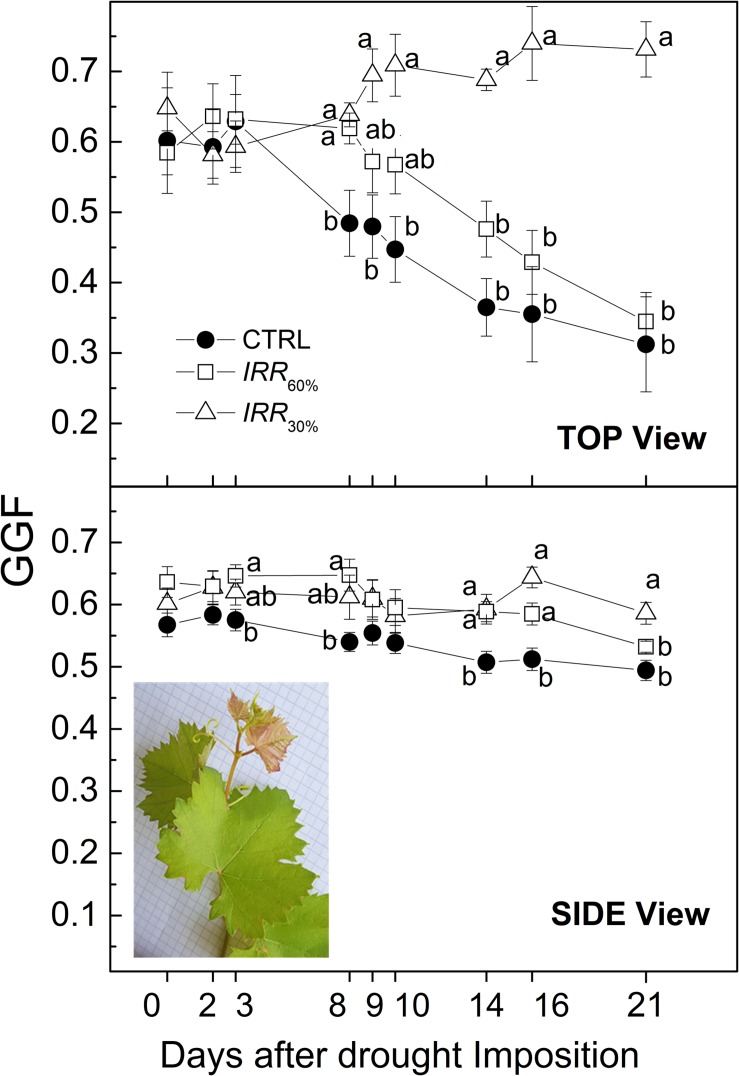
Evolution of Greener Fraction (GGF) recorded from top and side views throughout the experiment in vines well irrigated (CTRL) and under drought stress receiving 60% (*IRR*_60%_) and 30% (Δ, *IRR*_30%_) of maximum soil available water capacity. Comparing treatments at the same time different letters indicate statistically significant differences according to Tukey’s HSD test, *p* < 0.05. Note that letters were not reported when differences were not statistically significant. In the inset of the Side view panel a 1-year growing shoot of the Aleatico cv. (*Vitis vinifera*) showing typical copper-reddish young leaves pictured on a background with 0.5 cm mesh. Note that colors apply only for electronic version of the article.

Decreasing of the green color fraction (or increasing yellow or brown ones) is usually associated to onset and progress of leaf senescence as induced also by drought to the extent that reduction or prolonged persistence of green color are thought robust indicators to discriminate drought prone or tolerant plants (Munné-Bosch and Alegre, 2004; Cai et al., 2016; Duan et al., 2018). During the early 3 DADI, GGF values were not statistically significant among the irrigation treatments, showing a GGF sitting at around 0.6 (Figure 10). Starting from 8 DADI, although Ψ was stable the GGF progressively decreased in CTRL and *IRR*_60%_ vines. At the end of the experiment (21 DADI) both CTRL and *IRR*_60%_ plants scored a statistically significant difference in GGF, approximately 50% less than that found in most drought stressed vines (*IRR*_30%_). By contrast, TOP view GGF of *IRR*_30%_ increased from 0.6 (8 DADI) to 0.72 after 13 days of exposure to drought.

The GGF index has been conceptualized to track senescence (i.e., the more GGF is stable, the less senescence occurs) (Casadesús et al., 2007; Casadesús and Villegas, 2014). The variation of GGF across treatments as a response to drought was influenced by the position of the RGB camera (side or top). When vines were imaged Side view the GGF of CRTL and *IRR*_60%_ treatments showed a similar 12–15% decreasing trend throughout the experiment, while *IRR*_30%_ was substantially stable if a transient increase at 16 DADI is excepted (Figure 10). Top view images reveal an approximately 50% decline of GGF in CTRL and *IRR*_60%_ at the end of experiment, by contrast in *IRR*_30%_ vines increased by approximately 22% (Figure 10).

Reduction of GGF values in both CTRL and *IRR*_60%_ vines during the advancement of drought (Figure 10) suggests an apparent progressing senescence particularly in case of top view. However, considering that both CTRL and *IRR*_60%_ vines continued to grow during the experiment (Figure 9) reduction of GGF might be an artifact related to the increased of not-green leaf area due to the emerging new leaves. Typically young leaves are copper-reddish colored in Aleatico cv. according to internationally recognized ampelographic description (Figure 10; IPGRI et al., 1997; Organisation Internationale de la Vigne et du Vin [OIV], 2009). Calculation of the GGF has been plausibly influenced by such new (non-green) even small sized foliage that increased the total pixels. The gentler decline of GGF derived from side view images compared to top ones might be explained considering that new emerging copper-reddish leaves (belonging the shoot apex) conceivably represented a smaller fraction of the pictured canopy. Hence, application of GGF index to track leaf senescence in grapevine should be cautiously used in relation to color-related juvenile traits of cultivars. An alternative or integrated GGF index accounting for possible dichromatism of young leaves is desirable.

The increase of GGF detected top view in *IRR*_30%_ vines was likely due to increased chloroplast density per unit leaf area as reported for barley (Munns et al., 2010). The GGF accounts for greener pixels hence it might be considered an analogous of the Dark (or deep) Green fraction (Cai et al., 2016). The pattern of GGF observed in this study during the progress of the drought stress apparently differs from that of the Dark Green fraction reported in Briglia et al. (2019) for the same Ψ typology (i.e., midday) and range (−0.4/−1.1 MPa). Particularly, the greener fraction in Briglia et al. (2019) (i.e., Dark Green) measured in severely drought stressed vines decreased with worsening of drought while in the present study the analogous GGF was stable (side view) or even increased (top view) (Figure 10). Growing conditions (e.g., temperature, light level) differed between the HTP facilities belonging Briglia et al. (2019) and that used this study. For example, irradiance was set at 800 μmol m^–2^ s^−1^ in this study while it followed the diurnal change in Briglia et al. (2019) being on average 1,350 μmol m^–2^ s^−1^ and often peaking at approximately 2,000 μmol m^–2^ s^−1^. In addition, Briglia et al. (2019) used ownrooted vines while in this study vines were grafted on a rootstock. Different irradiance and plant material possibly have influenced nutrient uptake/partitioning, leaf pigments concentration, thickness and in turn its RGB response and the course of senescence (Munné-Bosch and Alegre, 2004). It emerges the need for monitoring and setting of standard environmental growing conditions toward standardization of data acquisition as debated by the plant phenotyping community under several international initiatives (Pieruschka and Schurr, 2019; Rosenqvist et al., 2019).

## Conclusion

This paper demonstrates that 3D image-based leaf angle phenotyping is a promising tool to estimate plant water status across a wide range of drought stress. The present results document the close correspondence of leaf angle with leaf water potential in grapevines and indicate that imaging, although less well correlated with water potential than manual measurements, provides the opportunity to scale analysis at low cost. This study also documents the suitability of an image-based leaf area estimation model across two HTP plant phenotyping facilities. The study shows that for the suitability of green-related indices (e.g., GGF, Dark Green) for comparisons across platforms and the uniformity of plant material and possible cv-related trait (e.g., dichromatism of young leaves) and growing conditions would be required.

## Data Availability Statement

The datasets generated for this study are available on request to the corresponding author.

## Author Contributions

NB, GM, AP, VN, and JD conceptualized the experiment. FC and KW provided the technical assistance including image capture and plant treatments. NB and KW carried out the phenotyping, physiological activities, and the 2D-imaging data analyses. NB and DA carried out the physiological data analyses and interpretations. WY, DW YL ST, and WY performed the 3D modeling and analysis. NB, GM, and DA wrote the manuscript. AP, FC, VN, and JD reviewed the manuscript. VN, FC, and JD obtained the funding and JD provided access to the facilities. All authors have read and approved the final manuscript.

## Conflict of Interest

The authors declare that the research was conducted in the absence of any commercial or financial relationships that could be construed as a potential conflict of interest.

## Footnotes

1 https://www.plant-phenomics.ac.uk/index.php/resources/methodology/

2 https://github.com/NPPC-UK/Gravimetrics

## Supplementary Material

VIDEO S1 Animated 3D point clouds reconstruction of a grapevine plant using multi-view stereo method.

## References

[B1] Bailey B. N. Mahaffee W. F. (2017). Rapid measurement of the three-dimensional distribution of leaf orientation and the leaf angle probability density function using terrestrial LiDAR scanning. *Remote Sens. Environ.* 194 63–76. 10.1016/j.rse.2017.03.011

[B2] Berger B. Parent B. Tester M. (2010). High-throughput shoot imaging to study drought responses. *J. Exp. Bot.* 61 3519–3528. 10.1093/jxb/erq201 20660495

[B3] Biskup B. Scharr H. Schurr U. Rascher U. W. E. (2007). A stereo imaging system for measuring structural parameters of plant canopies. *Plant Cell Environ.* 30 1299–1308. 17727419 10.1111/j.1365-3040.2007.01702.x

[B4] Black C. A. (1965). *Methods of Soil Analysis: Part I Physical and Mineralogical Properties.* Madison, WA: American Society of Agronomy.

[B5] Boso S. Alonso-Villaverde V. Santiago J. L. Gago P. Dürrenberger M. Düggelin M. (2010). Macro-and microscopic leaf characteristics of six grapevine genotypes (*Vitis* spp.) with different susceptibilities to grapevine downy mildew. *Vitis* 49 43–50.

[B6] Briglia N. Montanaro G. Petrozza A. Summerer S. Cellini F. Nuzzo V. (2019). Drought phenotyping in Vitis vinifera using RGB and NIR imaging. *Sci. Hortic.* 15:108555.

[B7] Cai J. Okamoto M. Atieno J. Sutton T. Li Y. Miklavcic S. J. (2016). Quantifying the onset and progression of plant senescence by color image analysis for high throughput applications. *PloS One* 11:e0157102. 10.1371/journal.pone.0157102 27348807 PMC4922665

[B8] Campo M. G. D. Ruiz C. Sotés V. Lissarrague J. (1999). Water consumption in grapevines: influence of leaf area and irrigation. *Acta Hortic.* 526 193–200. 10.17660/actahortic.1999.493.27

[B9] Carvalho L. C. Vidigal P. Amâncio S. (2015). Oxidative stress homeostasis in grapevine (*Vitis vinifera* L.). *Front. Environ. Sci.* 3:20 10.3389/fenvs.2015.00020

[B10] Casadesús J. Kaya Y. Bort J. Nachit M. M. Araus J. L. Amor S. (2007). Using vegetation indices derived from conventional digital cameras as selection criteria for wheat breeding in water-limited environments. *Ann. Appl. Biol.* 150 227–236.

[B11] Casadesús J. Villegas D. (2014). Conventional digital cameras as a tool for assessing leaf area index and biomass for cereal breeding. *J. Integrat. Plant Biol.* 56 7–14. 10.1111/jipb.12117 24330531

[B12] Chaves M. M. Zarrouk O. Francisco R. Costa J. M. Santos T. Regalado A. P. (2010). Grapevine under deficit irrigation: hints from physiological and molecular data. *Ann. Bot.* 105 661–676. 20299345 10.1093/aob/mcq030 PMC2859908

[B13] Chone X. Van Leeuwen C. Dubourdieu D. Gaudillère J. P. (2001). Stem water potential is a sensitive indicator of grapevine water status. *Ann. Bot.* 87 477–483.

[B14] Cifre J. Bota J. Escalona J. M. Medrano H. Flexas J. (2005). Physiological tools for irrigation scheduling in grapevine (*Vitis vinifera* L.). *Agric. Ecosyst. Environ.* 106 159–170. 10.1016/j.agee.2004.10.005

[B15] Das Choudhury S. Samal A. Awada T. (2019). Leveraging image analysis for high-throughput plant phenotyping. *Front. Plant Sci.* 10:508. 10.3389/fpls.2019.00508 31068958 PMC6491831

[B16] de Mendiburu F. (2016). *Agricolae: Statistical Procedures for Agricultural Research. R Package Version 1.2-4*. Available online at: https://cran.r-project.org/package=agricolae

[B17] Duan L. Han J. Guo Z. Tu H. Yang P. Zhang D. (2018). Novel digital features discriminate between drought resistant and drought sensitive rice under controlled and field conditions. *Front. Plant Sci.* 9:492. 10.3389/fpls.2018.00492 29719548 PMC5913589

[B18] Fernández J. E. (2014). Plant-based sensing to monitor water stress: applicability to commercial orchards. *Agric. Water Manag.* 142 99–109.

[B19] Fernández J. E. (2017). Plant-based methods for irrigation scheduling of woody crops. *Horticulturae* 3:35 10.3390/horticulturae3020035

[B20] Gago J. Fernie A. R. Nikoloski Z. Tohge T. Martorell S. Escalona J. M. (2017). Integrative field scale phenotyping for investigating metabolic components of water stress within a vineyard. *Plant Methods* 13:90. 10.1186/s13007-017-0241-z PMC5663058 29093742

[B21] Gamon J. A. Pearcy R. W. (1989). Leaf movement, stress avoidance and photosynthesis in *Vitis* californica. *Oecologia* 79 475–481. 28313481 10.1007/BF00378664

[B22] Giorio P. Nuzzo V. (2012). Leaf area, light environment, and gas exchange in Montepulciano grapevines trained to Tendone trellising system. *Plant Biosyst.* 146 322–333. 10.1080/11263504.2011.557095

[B23] Goldhamer D. Fereres E. (2001). Irrigation scheduling protocols using continuously recorded trunk diameter measurements. *Irri. Sci.* 20 115–125. 10.1007/s002710000034

[B24] Goudriaan J. van Laar H. H. (1994). “Development and growth,” in *Modelling Potential Crop Growth Processes Current Issues in Production Ecology*, (Dordrecht: Springer), 69–94.

[B25] Greer D. H. Weedon M. M. (2011). Modelling photosynthetic responses to temperature of grapevine (*Vitis vinifera* cv. *Semillon*) leaves on vines grown in a hot climate. *PlantCell Environ.* 35 1050–1064. 10.1111/j.1365-3040.2011.02471.x 22150771

[B26] Hochberg U. Degu A. Fait A. Rachmilevitch S. (2013). Near isohydric grapevine cultivar displays higher photosynthetic efficiency and photorespiration rates under drought stress as compared with near anisohydric grapevine cultivar. *Physiol. Plant.* 147 443–452. 10.1111/j.1399-3054.2012.01671.x 22901023

[B27] IPGRI, UPOV, and OIV (1997). *Descriptors for Grapevine (Vitis spp.).* Geneva: International Union for the Protection of New Varieties of Plants.

[B28] Jones H. G. (2006). Monitoring plant and soil water status: established and novel methods revisited and their relevance to studies of drought tolerance. *J. Exp. Bot.* 58 119–130. 10.1093/jxb/erl118 16980592

[B29] Kao W. Y. Forseth I. N. (1991). The effects of nitrogen, light and water availability on tropic leaf movements in soybean (*Glycine max*). *Plant Cell Environ.* 14 287–293. 10.1111/j.1365-3040.1991.tb01503.x

[B30] Kao W. Y. Forseth I. N. (1992). Dirunal leaf movement, chlorophyll fluorescence and carbon assimilation in soybean grown under different nitrogen and water availabilities. *Plant Cell Environ.* 15, 703–710. 10.1111/j.1365-3040.1992.tb01012.x

[B31] Khanna A. Kaur S. (2019). Evolution of Internet of Things (IoT) and its significant impact in the field of precision agriculture. *Comput. Electron. Agric.* 157 218–231. 10.1016/j.compag.2018.12.039

[B32] Lampinen B. Shackel K. Southwick S. Olson W. Dejong T. (2004). Leaf and canopy level photosynthetic responses of French prune (*Prunus domestica* L. ‘French’) to stem water potential based deficit irrigation. *J. Hortic. Sci. Biotechnol.* 79 638–644. 10.1080/14620316.2004.11511819

[B33] Lou L. Liu Y. Han J. Doonan J. H. (2014). “Accurate multi-view stereo 3d reconstruction for cost-effective plant phenotyping,” in *Image Analysis and Recognition. ICIAR 2014. Lecture Notes in Computer Science*, Vol. 8815 eds Campilho A. Kamel M. (Cham: Springer).

[B34] Louarn G. Lecoeur J. Lebon E. (2007). A Three-dimensional statistical reconstruction model of grapevine (*Vitis vinifera*) simulating canopy structure variability within and between cultivar/training system pairs. *Ann. Bot.* 101 1167–1184. 10.1093/aob/mcm170 18202006 PMC2710267

[B35] Maloof J. N. Nozue K. Mumbach M. R. Palmer C. M. (2013). LeafJ: an ImageJ plugin for semi-automated leaf shape measurement. *J. Vis. Exp.* 71:e50028. 10.3791/50028 23380664 PMC3582691

[B36] Medrano H. Escalona J. M. Cifre J. Bota J. Flexas J. (2003). A ten-year study on the physiology of two Spanish grapevine cultivars under field conditions: effects of water availability from leaf photosynthesis to grape yield and quality. *Func. Plant Biol.* 30 607–619. 10.1071/FP02110 32689046

[B37] Minasny B. McBratney A. B. (2003). Integral energy as a measure of soil-water availability. *Plant Soil* 1 253–262.

[B38] Munné-Bosch S. Alegre L. (2004). Die and let live: leaf senescence contributes to plant survival under drought stress. *Funct. Plant Biol.* 31 203 10.1071/fp03236 32688892

[B39] Munns R. James R. A. Sirault X. R. R. Furbank R. T. Jones H. G. (2010). New phenotyping methods for screening wheat and barley for beneficial responses to water deficit. *J. Exp. Bot.* 6 3499–3507. 10.1093/jxb/erq199 20605897

[B40] Norman J. M. Campbell G. S. (1989). “Canopy structure,” in *Plant Physiological Ecology*, eds Pearcy R. W. Ehleringer J. R. Mooney H. A. Rundel P. W. (Springer: Dordrecht), 301–325. 10.1007/978-94-009-2221-1_14

[B41] Organisation Internationale de la Vigne et du Vin [OIV] (2009). *(International Organisation of Vine & Wine), Description of World Vine Varieties.* Avaliable at: http://www.oiv.int/public/medias/2272/des-cep-monde-edition-2009.pdf (accessed November, 2019).

[B42] Ortuño M. Conejero W. Moreno F. Moriana A. Intrigliolo D. Biel C. (2010). Could trunk diameter sensors be used in woody crops for irrigation scheduling? A review of current knowledge and future perspectives. *Agric. Water Manag.* 97 1–11. 10.1016/j.agwat.2009.09.008

[B43] Palliotti A. Silvestroni O. Petoumenou D. Vignaroli S. Berrios J. G. (2008). Evaluation of low-energy demand adaptive mechanisms in Sangiovese grapevine during drought. *OENO One* 42 41–47. 10.20870/oeno-one.2008.42.1.832

[B44] Paolocci M. Muganu M. Alonso-Villaverde V. Gindro K. (2014). Leaf morphological characteristics and stilbene production differently affect downy mildew resistance of Vitis vinifera varieties grown in Italy. *Vitis* 53 155–161.

[B45] Paulus S. Dupuis J. Mahlein A. K. Kuhlmann H. (2013). Surface feature based classification of plant organs from 3D laserscanned point clouds for plant phenotyping. *BMC Bioinformatics* 14:238. 10.1186/1471-2105-14-238 23890277 PMC3750309

[B46] Pieruschka R. Schurr U. (2019). Plant phenotyping: past, present, and future. *Plant Phen.* 2019 1–6. 10.34133/2019/7507131 PMC7718630 33313536

[B47] Raymond F. Ullmann A. Tramblay Y. Drobinski P. Camberlin P. (2019). Evolution of mediterranean extreme dry spells during the wet season under climate change. *Reg. Environ. Change* 19 2339–2351. 10.1007/s10113-019-01526-3

[B48] Reynolds D. Baret F. Welcker C. Bostrom A. Ball J. Cellini F. (2019). What is cost-efficient phenotyping? Optimizing costs for different scenarios. *Plant Sci.* 282 14–22. 10.1016/j.plantsci.2018.06.015 31003607

[B49] Rosenqvist E. Großkinsky D. K. Ottosen C. O. Zedde R. V. D. (2019). The phenotyping dilemma—the challenges of a diversified phenotyping community. *Front. Plant Sci.* 10:163. 10.3389/fpls.2019.00163 30873188 PMC6403123

[B50] Schindelin J. Arganda-Carreras I. Frise E. (2012). Fiji: an open-source platform for biological-image analysis. *Nat. Methods* 9 676–682. 10.1038/nmeth.2019 22743772 PMC3855844

[B51] Schurr U. Walter A. Rascher U. (2006). Functional dynamics of plant growth and photosynthesis – from steady-state to dynamics – from homogeneity to heterogeneity. *PlantCell Environ.* 29 340–352. 10.1111/j.1365-3040.2005.01490.x 17080590

[B52] Shackel K. A. (2007). Water relations of woody perennial plant species. *OENO One* 41 121–129.

[B53] Smart R. E. (1974). Aspects of water relations of the grapevine (*Vitis vinifera*). *Am. J. Enol. Vitic.* 25 84–91.

[B54] Srivastava S. Bhugra S. Lall B. Chaudhury S. (2017). “Drought stress classification using 3D plant models,” in *2017 IEEE International Conference on Computer Vision Workshops* (Venice: ICCVW), 2046–2054.

[B55] Torrecillas A. Domingo R. Galego R. Ruiz-Sánchez M. (2000). Apricot tree response to withholding irrigation at different phenological periods. *Sci. Hortic.* 85 201–215. 10.1016/s0304-4238(99)00146-6

[B56] Turner N. C. (1981). Techniques and experimental approaches for the measurement of plant water status. *Plant Soil* 58 339–366.

[B57] Vicari M. B. Pisek J. Disney M. (2019). New estimates of leaf angle distribution from terrestrial LiDAR: comparison with measured and modelled estimates from nine broadleaf tree species. *Agric. For. Meteorol.* 264 322–333. 10.1016/j.agrformet.2018.10.021

[B58] Wu C. (2013). “Towards linear-time incremental structure from motion,” in *2013 International Conference on 3D Vision-3DV*, Piscataway, NJ: IEEE, 127–134.

[B59] Xiong X. Yu L. Yang W. (2017). A high-throughput stereo-imaging system for quantifying rape leaf traits during the seedling stage. *Plant Methods* 13 7. 10.1186/s13007-017-0157-7 28163771 PMC5282657

[B60] Yıldırım K. Yaðcı A. Sucu S. Tunç S. (2018). Responses of grapevine rootstocks to drought through altered root system architecture and root transcriptomic regulations. *Plant Physiol. Biochem.* 127 256–268. 29627732 10.1016/j.plaphy.2018.03.034

[B61] Zaman-Allah M. Vergara O. Araus J. L. Tarekegne A. Magorokosho C. Zarco-Tejada P. J. (2015). Unmanned aerial platform-based multi-spectral imaging for field phenotyping of maize. *Plant Methods* 24:35. 10.1186/s13007-015-0078-2 PMC4477614 26106438

[B62] Zhao C. Zhang Y. Du J. Guo X. Wen W. Gu S. (2019). Crop phenomics: current status and perspectives. *Front. Plant Sci.* 10:714. 10.3389/fpls.2019.00714 31214228 PMC6557228

[B63] Zhu J. Dai Z. Vivin P. Gambetta G. A. Henke M. Peccoux A. (2018). A 3-D functional–structural grapevine model that couples the dynamics of water transport with leaf gas exchange. *Ann. Bot.* 18 833–848. 10.1093/aob/mcx141 PMC5906973 29293870

